# Whole Genome Analysis in Consanguineous Families Reveals New Loci for Speech Sound Disorder (SSD)

**DOI:** 10.3390/genes15081069

**Published:** 2024-08-13

**Authors:** Tahira Yasmin, Aatika Sadia, Laraib Nadeem, Muhammad Asim Raza Basra, Mabel L. Rice, Muhammad Hashim Raza

**Affiliations:** 1 Centre for Clinical and Nutritional Chemistry, School of Chemistry, University of the Punjab, Lahore 54590, Pakistan; tahira3100@gmail.com (T.Y.); aatiasj@gmail.com (A.S.); laraibnadeem321@gmail.com (L.N.); asimbasra.chem@pu.edu.pk (M.A.R.B.); 2 Laboratory of Organic Electronics, Department of Science and Technology, Linköping University, Norrköping Campus, 60221 Norrköping, Sweden; 3 Speech-Language-Hearing Sciences & Disorders, University of Kansas, Lawrence, KS 66045, USA; mabel@ku.edu

**Keywords:** speech sound disorder, articulation, family-based linkage analysis, homozygosity mapping, Pakistani families

## Abstract

Speech is the most common means of communication in humans. Any defect in accurate speech production ability results in the development of speech sound disorder (SSD), a condition that can significantly impair an individual’s academic performance, social interactions, and relationships with peers and adults. This study investigated the genetic basis of SSD in three Pakistani families. We performed family-based genome-wide parametric linkage analysis and homozygosity mapping in three consanguineous families with SSD from the Punjab province of Pakistan. The Test for Assessment of Articulation and Phonology in Urdu (TAAPU) was used to analyze the speech articulation data and determine the Percentage Correct Consonants (PCC) score. The PCC score defined the affected and unaffected individuals in each family. Parametric linkage analysis revealed a linkage to chromosome 5 (5q21.3-5q23.1) with a significant logarithm of the odds (LOD) score of 3.13 in a Pakistani family with specific language impairment-97 (PKSLI-97) under an autosomal recessive mode of inheritance. The other two families showed a suggestive linkage at 6p22.1, 14q12, and 16q12.1 under the recessive mode of inheritance. Interestingly, homozygosity mapping showed a loss of heterozygosity in the linkage region at 5q15-5q23.1, shared among seven affected (mostly in the younger generation) and one unaffected individual of PKSLI-97. Our analysis identified the 6p22 locus previously implicated in dyslexia, childhood apraxia of speech (CAS), and language impairment, confirming the role of KIAA0319 and DCDC2 in this locus. These findings provide statistical evidence for the genomic regions associated with articulation disorder and offer future opportunities to further the role of genes in speech production.

## 1. Introduction

Speech sound disorder (SSD), also known as articulation disorder, is a developmental disorder characterized by the combination of different problems of speech. According to the American Speech–Language–Hearing Association (ASHA), “SSD is an umbrella term referring to any difficulty or combination of problems with perception, motor production, or phonological representation of speech sounds and speech segments, including phonotactic rules governing permissible speech sound sequences in a language” [1,2]. Individuals with SSD are significantly less skilled than typically developing individuals in articulating, sequencing, or organizing speech sounds without any apparent etiologies such as cognitive, sensory, and neuro-motor problems [3,4]. Individuals with SSD may have a difficulty or a combination of issues like omissions, substitutions, or distortions and the addition of phonemes within words that may impact speech intelligibility and acceptability [5]. Children with SSD struggle with spoken language production; more than half of these children experience difficulties with later literacy skills [6,7]. The effects of SSD may be lifelong and the biological basis of this disorder is primarily unknown [6]. Recent studies supported the involvement of genetic factors in SSD, which hold promise in expanding our knowledge of underlying mechanisms [1,8,9,10].

The prevalence of SSD estimated in primary school-going children (age 5–6 years) in the Punjab province of Pakistan is 1.3% [11], while in English-speaking preschool children in the United States is 15.6% [12], which reduces to 3.8% in 6-year-olds. The worldwide prevalence of SSD varies (1 to 17%) from one population to another [11,13,14,15,16]. Furthermore, the prevalence of SSD is higher in males than in females. Like in English and Persian-speaking children of age 6, SSD was 4.5% and 17.4% in males and 3.1% and 9.1% in females, respectively [13,15]. Similarly, in primary school-going children in Pakistan, a higher prevalence of SSD in males was observed than in females, with a male-to-female ratio of 1:1.26 [11]. SSD is typically diagnosed and treated in childhood; therefore, the prevalence rate is much higher in younger children as compared to adults. It is rarely seen in adolescents and adults and prevalence rates of persistent SSD are 1–2% in adults [17].

Language impairment (LI) has been defined as various deficits in developmental expressive and receptive language with normal hearing [18]. The comorbidity of language impairment has been reported in children with SSD [19]. About 5 to 8% of children with Specific Language Impairment (SLI) showed speech difficulties [13,20]. In children with comorbid SSD and LI phenotype, an expressive language disorder (38–62%) is three times more prevalent than a receptive language disorder (6–21%) [13,19]. Children with LI and SSD experienced more difficulties in academic performance, showing poor performance in different measures of phonological representations, phonological memory, vocabulary, reading decoding, spelling, reading comprehension, and other language skills compared to children with SSD only [21,22]. 

Speech articulation abilities of individuals with SSD are assessed through standardized measures of speech. The Goldman-Fristoe Test of Articulation (GFTA) is a standardized test commonly used to measure the articulation and production of consonant sounds in English-speaking populations [23]. Similarly, a locally developed standardized assessment tool, a Test for Assessment of Articulation and Phonology in Urdu (TAAPU), measures articulation/speech production errors in Urdu-speaking populations in Pakistan [24]. 

Twin studies provided the highest heritability estimates (up to 0.98) for SSD depending upon the endophenotypes considered [25,26,27,28]. Familial aggregation studies reported the presence of a higher percentage of family members with impaired speech in families than in the control population [29]. In a familial aggregation study, approximately 26% of first-degree relatives and 13.7% of extended family members with speech difficulty were observed. This study showed that a higher percentage of males were affected (40.9%) than females (19.4%) [30]. Familial aggregation and high heritability estimates indicate that genetic components are involved in speech and language disorders [9].

Several studies utilized the loss of heterozygosity method and identified numerous risk genes for inherited disorders like specific language impairment (SLI), autism, and Intellectual Disability [31,32,33,34]. In a genetic study on the Chilean population with a high frequency of language impairment, linkage analysis and loss of heterozygosity identified linkage to 7q [35,36]. Recently, loss of heterozygosity and family-based linkage analysis in consanguineous and outbred families from Pakistan and the United States identified new SLI loci and confirmed the previous findings [32,37].

The current study aimed to perform the genetic analysis in three consanguineous families with SSD ascertained from Pakistan. 

## 2. Materials and Methods

### 2.1. Identification and Enrollment of Families

**Figure 1:**
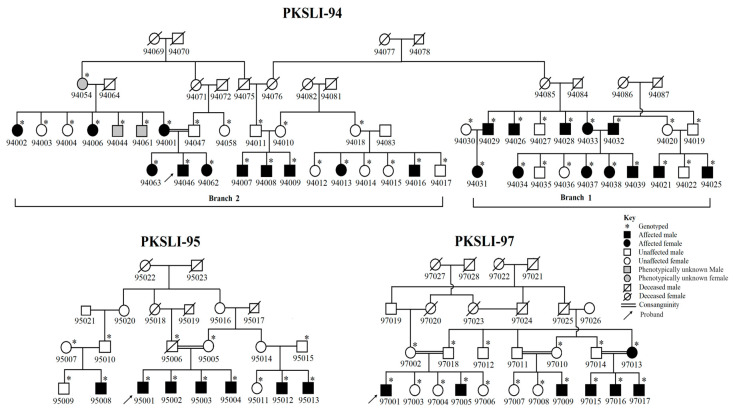
Pedigrees of Pakistani families with speech sound disorder. PKSLI-94 is a large family divided into two branches. The square represents males and the circle represents females. Black-filled symbols showed SSD-affected individuals, unfilled symbols showed unaffected individuals, and gray-filled symbols showed phenotypically unknown individuals. The diagonal line across the symbol shows deceased individuals. * represents the genotyped individuals and the double marriage line represents consanguinity. Each individual is assigned a unique ID (five digits), starting with the family number and then the individual number.

For this study, three extended families with speech articulation history were selected from an ongoing cohort of the genetics of specific language impairment (SLI). The families in this cohort were labeled as Pakistani families with specific language impairment (PKSLI); we adopted these family labels for the current study. In this cohort, a family history questionnaire developed by Mable L. Rice was used to gather the family history of speech and language impairment from the probands of age 10 to 17 years in each family. Based on the speech impairment history in probands, we followed up with three PKSLI families (PKSLI-94, PKSLI-95, and PKSLI-97) and obtained speech articulation data using a locally developed standardized measure for the Pakistani population, namely the Test for Assessment of Articulation and Phonology in Urdu (TAAPU) test, and we defined an SSD based on this test. These families belonged to Punjab, Pakistan, where caste-based and cousin marriages are common. The PKSLI-94 is a large family with 44 individuals, out of which 22 were affected with SSD (Figure 1). For genetic analysis, this family was divided into two branches, PKSLI-94-Branch 1 and PKSLI-94-Branch 2. In PKSLI-95, a TAAPU assessment was performed on 15 individuals, out of which 7 were affected with SSD. In PKSLI-97, TAAPU defined SSD in 7 out of 17 individuals. 

### 2.2. Collection and Evaluation of Speech Data

The speech data of all the available individuals in each family included in this study were collected using a standardized norm-referenced test of single-word productions, the TAAPU test. It consists of thirty-four phonemes used to name the sixty colorful pictures and a response sheet containing a word list of those pictures [24]. The TAAPU test provides information about speech articulation errors (substitution, omission, and addition errors) within phonemes at the initial, middle, and final positions within a word. When a participant produces a different sound and/or omits the sound rather than the actual phoneme sound, it is marked as an error. The errors may be substitution, omission, and addition of phonemes at the beginning, middle, and final position. At the end of the participant’s speech analysis, all errors were added up to make a total error. The Percentage of Correct Consonants (PCC) score was calculated using a word list in the TAAPU test. The PCC score was determined using the following formula [38]:

PCC = (Correct consonants/Total Consonants) × 100

The speech sound data were collected in a quiet room to ensure complete silence and no background noise while conducting this assessment using an audio recorder and/or smartphone. The recordings were examined/and analyzed by three trained graduate students with the help of a local speech–language pathologist (SLP) to note down articulation errors of substitution, omission, and addition [38]. The SLP trained three graduate students on how to analyze speech data carefully. Each individual’s PCC score was calculated using a previously used and reported formula [21]. The three PKSLI families are bilingual and speak Punjabi (regional language) and Urdu (national language). Bilingual children showed lower speech intelligibility and more speech errors than monolingual children [39]. The previously reported PCC score for typically developing bilinguals is 89 [40]. Therefore, individuals who obtained PCC scores less than 89 were defined as affected with SSD and individuals with PCC scores equal to and/or greater than 89 were described as unaffected [40,41]. We used this assessment scale for TAAPU to define the affected and unaffected individuals in each family. 

### 2.3. DNA Extraction and Whole-Genome SNP Genotyping

Blood/saliva samples were obtained from the available family members for DNA. A blood sample (5 mL) was collected into an anticoagulant (2% EDTA solution; 500 µL for 5 mL blood sample) containing 50 mL falcon tubes and stored at −20 °C. A saliva sample of up to 2 mL was collected in an Oragene-Discover OGR-500 Kit from DNA Genotek (Oragene) stored at room temperature. According to the manufacturer’s protocol, DNA was extracted from blood samples using an organic method and from saliva samples [42]. DNA samples of 78 individuals from 3 families were genotyped using Illumina Infinium QC Array-24 and the genotyping was performed at the Johns Hopkins University School of Medicine, Genetic Resources Core Facility [43]. This SNP array is highly cost-effective with low density and has proven effective in detecting ethnicity, sample-specific variant calls, consanguinity in samples, and gender [44]. It has been widely used in association studies and proved efficient enough to find genetic associations [32,37,44,45]. There are 15,949 SNPs in this array evenly distributed throughout the genome with an average density of 0.5 centi Morgan (cM), a unit to measure the frequency of chromosomal recombination. Out of 15,949 SNPs, autosomal chromosomes contain 11,994 SNPs and the rest spread across mitochondrial and sex chromosomes. GenomeStudio v 2.0 was used to extract the SNP genotyping data and perform clustering and quality control [46]. There was 0.11% missing data and the subset of SNPs was manually reviewed, including SNPs with cluster separation < 0.4, AB T Dev ≥ 0.065, AA R Mean < 0.3, AB R Mean < 0.3, BB R Mean < 0.3, or a call rate < 99%. After quality control measures, 49 SNPs were excluded and a total of 11,925 SNPs were used for genetic analysis, i.e., linkage analysis and homozygosity mapping.

### 2.4. Linkage Analysis

Genome-wide parametric linkage analysis was performed using SUPERLINK ONLINE SNP 1.1 (http://cbl-hap.cs.technion.ac.il/superlink-snp/, accessed on 17 May 2022). The linkage analysis was carried out under a recessive mode of inheritance with complete disease penetrance (0.99) and the allele frequency was set to 0.001. The PKSLI-94 was divided into two branches, PKSLI-94 (Branch 1 and Branch 2), due to their distant relatedness, and the genetic analysis was performed in each unit independently. Whereas, the other two families, PKSLI-95 and PKSLI-97, were analyzed as a whole. According to previous studies, thresholds of LOD score for suggestive and significant linkage were 1.5 and 3.0, respectively, in parametric linkage analysis. A LOD score of −2 or less is reflected as an indication against linkage [47,48]. 

### 2.5. Homozygosity Mapping

Homozygous regions in the genome are defined as long stretches of homozygous genotypes inherited together as haplotypes [49]. Long homozygous regions in family-based genetic studies of inherited disorders may carry a segregating variant allele and indicate a recessive mode of inheritance [31]. Homozygosity mapping was performed in all families using a homozygosity mapper (https://www.homozygositymapper.org/, accessed on 4 June 2022) that can identify the stretches of the homozygous blocks across the genome [50]. A homogeneity option in the homozygosity mapper was selected and the disease allele frequency was set to zero to determine shared homozygous blocks among affected individuals. The stretch of the homozygous block was excluded if it contained more than 20 SNP markers in controls [50]. Consanguinity favors the extended homozygous region in a consanguineous population like Pakistan, whose consanguinity rate is approximately 62%. The homozygous regions of <1–4 Mb were frequently present (41.74%) in phenotypically characterized Pakistani controls [51]. Therefore, initially, we considered the homozygous regions >4 Mb and those present in two or more affected individuals and absent in the unaffected except for one unaffected individual. 

### 2.6. Haplotyping

**Figure 2:**
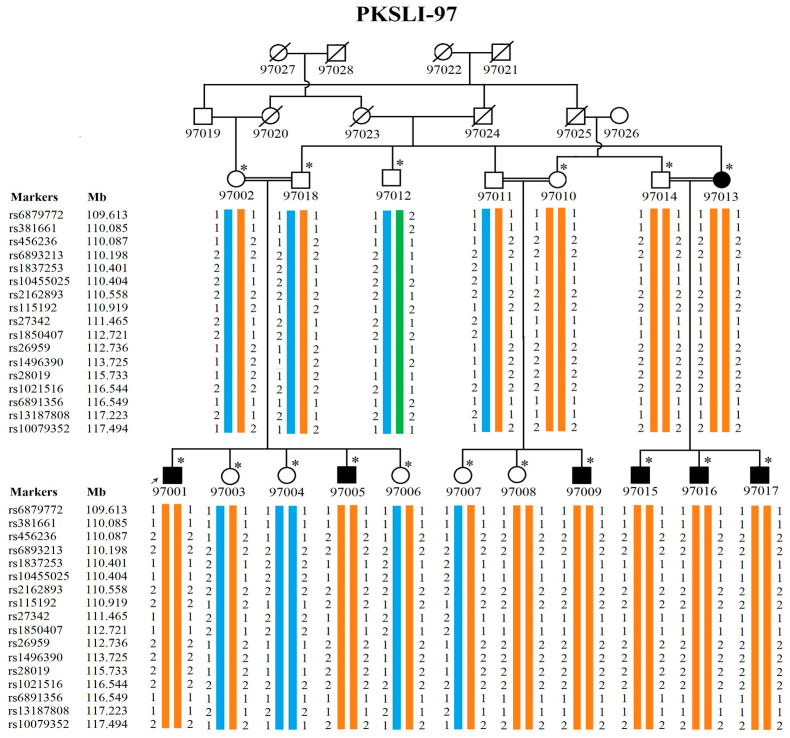
Haplotype in PKSLI-97 at chromosome 5q21.3-5q23.1. The square represents males and the circle represents females. Black-filled symbols showed SSD-affected individuals and unfilled symbols showed unaffected individuals. The diagonal line across the symbol shows deceased individuals. * represents the genotyped individuals and a double marriage line represents consanguinity. The markers and their chromosomal location are listed to the left of the two younger generations. The haplotypes drawn under each individual are represented with three colors. The diseased haplotype is labeled with orange and the wild type/normal haplotype is labeled with blue and green.

We manually constructed haplotypes in family PKSLI-97 for the linkage region on chromosome 5 (5q21.3-5q23.1) and drew the haplotypes in pedigree (Figure 2).

## 3. Results

### 3.1. Speech Articulation Analysis

Speech analysis revealed that substitution errors were more prevalent in the test participants than omission and addition errors (Table 1, Table 2 and Table 3).

Speech articulation analysis of PKSLI-94 (Branch 1) exhibited the highest substitution errors in three alphabets: خ [x], ک [k], and ڈ [ɖ]. Similarly, the highest omission errors were observed in alphabets ح [h] and خ [x]. We observed addition errors in only 6 out of 34 alphabets; thus, they are less prevalent in all individuals (Tables S1–S3). The individuals in PKSLI-94 (Branch 2) produced the highest substitution errors in four alphabets: خ [x], غ [γ], ک [k], and گ [g]. The omission errors in Branch 2 of PKSLI-94 were observed less frequently than in Branch 1. The highest omission errors were observed in the alphabet ح [h]. In Branch 2, we observed addition errors in only 7 of 34 alphabets. The highest addition errors were observed for alphabet ی [j/i] in all individuals of PKSLI-94 (Branch 2) (Tables S4–S6). Similarly, speech samples of PKSLI-95 showed the highest substitution errors in three alphabets: خ [x], ڑ [ɽ] and ڈ [ɖ]. The omission and addition errors were less prevalent in PKSLI-95 (Tables S7–S9). Speech articulation analysis of PKSLI-97 exhibits the highest substitution errors in four alphabets: ڈ [ɖ], ٹ [ʈ], ڑ [ɽ], and خ [x]. The omission and addition errors were less prevalent in PKSLI-97 (Tables S10–S12).

### 3.2. Linkage Analysis

We obtained the highest single-point LOD score of 3.13 on 5q21.3 in PKSLI-97 (Table 4). The SNP markers (rs1045706 and rs13157174) with negative LOD scores marked the linkage region boundaries, limiting the locus to 9 Mb on 5q21.3. The haplotype analysis on 5q21.3 showed all the affected individuals inherited the diseased haplotype (labeled with two orange chromosomes) (Figure 2). We observed the highest LOD score of 2.13 on 16q12.1 in Branch 1 of PKSLI-94 (Table 5). Linkage analysis did not reveal any region with a LOD score >1 in branch 2 or the whole PKSLI-94. We identified two suggestive linkage loci (6p22.1-p22.1 and 14q12-q21.1) with LOD scores of 2.13 and 2.80 in PKSLI-95 (Table 5). 

### 3.3. Homozygosity Mapping and Haplotype Analysis

**Figure 3:**
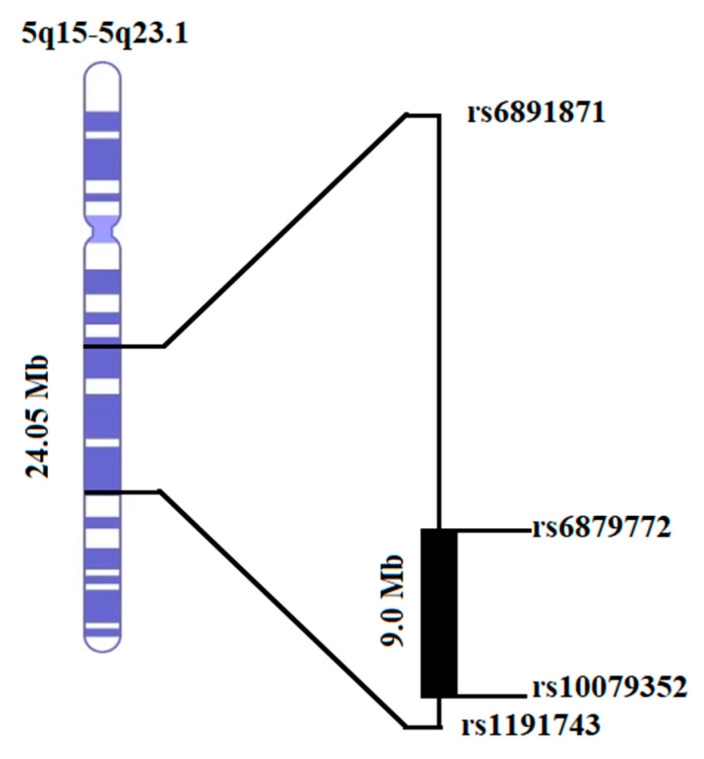
A homozygous region of 24.05 Mb on chromosome 5 was identified in the family PKSLI-97. Horizontal lines labeled with the markers show the proximal and distal boundaries of the homozygous region mentioned in Megabases (Mb). The filled rectangular area (9.0 Mb) represents the linked region found in PKSLI-97, with an LOD score of 3.13.

Using a homozygosity mapper, we found one homozygous region of 24.05 Mb on chromosome 5q15-5q23.1 in PKSLI-97 (Figure 3). Six affected individuals (97001, 97005, 97013, 97015, 97016, and 97017) shared the haplotype from rs6891871 to rs1191743. An unaffected individual (97004) was found to have a homozygous region but for the other allele (presented with blue color in Figure 2). Interestingly, the homozygous region (5q15-5q23.1) identified through homozygosity analysis overlapped with the linkage locus in PKSLI-97 (Figure 3). The homozygosity mapping in other families revealed no homozygous region shared among affected individuals.

## 4. Discussion

We used TAAPU and defined SSD in Pakistani families for genetic analysis. Using family-based parametric linkage analysis, in one family, we identified a new locus on chromosome 5 with a significant LOD score of 3.13, which was confirmed in this family independently by homozygosity mapping. In two other families, we identified three additional loci with suggestive LOD scores on chromosome 6p22, 14q12, and 16q12.1. 

The heterogeneity in SSD and sometimes shared phenotypic characteristics with other disorders, particularly reading disability (RD), SLI, and Attention Deficit/Hyperactivity Disorder (ADHD), indicates that SSD may share one or more genetic risk loci with different phenotypes [52]. Due to phenotypic comorbidity, researchers sometimes target the previously reported regions associated with RD and SLI and study these regions in families with SSD. Children with SSD and RD share phenotypic characteristics and may share the same loci with mutations in the same gene [1,53]. In multipoint linkage analysis, phonological memory showed strong linkage with microsatellite markers D3S2465 and D3S3716 with *p* values of 5.7 × 10^−5^ and 6.7 × 10^−4^, respectively. The Single-word decoding test (SWDT) was linked with markers D3S2465 and D3S1595 with *p* values of 0.004 and 0.005, respectively. A shared haplotype spanning D3S3049 and D3S3045 markers (4.9 centiMorgan (cM)) was observed in the sib pairs. These results confirmed the genetic influence on speech impairment and reading disorder [53]. Stein and colleagues targeted a region on chromosome 3 (DYX5; OMIM 606896) previously identified for RD [54]. Family-based non-parametric linkage analysis in 77 families with SSD showed a significant linkage at chromosome 3 with two traits: phonological decoding and phonological memory [53]. In another study, non-parametric linkage analysis was performed in 111 probands with a history of SSD. The analyses focused on previously known loci (1p36, 6p22.2, and 15q21) associated with RD, used a targeted linkage strategy, and found a suggestive linkage on chromosome 6p22.2 (OMIM 600202) and 15q21 (OMIM 127700) for two measures of speech [55]. Likewise, the effect of comorbidity in children with SSD and/or SLI was studied for the development of ADHD risks. A total of 108 children (ages 4 to 7) with and without SLI were enrolled and examined for the presence of ADHD symptoms. The results suggest that children in the SSD + SLI group exhibit higher levels of inattentive ADHD symptoms than those in the SSD-only and control groups [52]. Alternatively, it might be due to the involvement of one or more loci that overlap in SSD and ADHD, indicating the complex etiology of SSD [18,56]. Similarly, a case-control study on the Taiwanese Han population characterized a homozygous region on 11q22.3 in patients with an autism spectrum disorder. This region spanned several risk genes, including *NPAT* and *ATM*, which were interesting due to their role in speech delay and language impairment [33].

In PKSLI-97, we constructed haplotypes of the linked region on chromosome 5 (5q21.3-5q23.1). We observed two copies of the orange haplotype (causative haplotype) in all affected individuals and three phenotypically unaffected individuals (97010, 97014, and 97008). This might be explained by the fact that three unaffected individuals (two of them are the parents) could have a history of SSD who were recovered with the evidence that milder forms of SSD may recover independently or with the interventions [57]. As this family was initially recruited as an SLI family, we noticed that 97010 and 97008 are affected by language impairment. In the overall population of 5-year-old children, the cooccurrence of speech and language impairments was estimated at less than 2% [13]. We observed a similar level of comorbidity of SLI in family PKSLI-97, which is an interesting observation regarding the likelihood of shared genetic pathways. The literature showed that about 15% of children with persistent speech disorders show comorbidity of SLI and about 8% of children with SLI experience speech problems at age 6 [13]. With the help of haplotype analysis, we could find genetic causes in family PKSLI-97 that suggest the shared genotypes of these phenotypes. A common causative haplotype in individuals with SSD and SLI indicates a shared genetic basis for both impairments in this family. Another implication is reduced disease penetrance, which was noted before in other speech impairments, such as stuttering [58,59]. 

We investigated the candidate genes in 5q21.3-5q23.1 and found a few exciting genes for prospective studies. The *SLC25A46* is located in this region, which encodes a mitochondria solute carrier protein essential for mitochondrial fusion and mitochondrial lipid homeostasis [60,61]. The mutation in this gene was identified in North American families with cerebellar ataxia. The four affected individuals in three families showed the homozygous mutation in the *SLC25A46* with a consistent phenotype of childhood onset, optic atrophy, speech problems, and gait [62]. Other genes identified in this locus, like *CAMK4* and *KCNN2*, were involved in neuronal transmission [63,64]. The functional study in mice showed the role of CAMK4 in neuronal plasticity and memory. It was observed that activity-induced CREB phosphorylation and c-Fos expression significantly reduced, resulting in impaired Hippocampal late LTP (L-LTP). However, early LTP (E-LTP) and the primary synaptic role remain unaffected. These difficulties correlated with deficits in long-term memory, specifically in its retention level but not acquisition level [64]. The genetic variants in *KCNN2* were found in individuals with intellectual disability, developmental motor and language delays, and movement disorders. The animal models with spontaneous mutations in *KCNN2* also showed memory deficits [63]. Our previous study compared memory abilities and receptive vocabulary in children with SSD and typically developing peers. We identified that children with SSD have reduced short-term memory (STM), working memory (WM), and receptive vocabulary when compared with controls of the same age [38]. These genes might be helpful to explain the underlying memory deficits in individuals with SSD. 

We identified a suggestive linkage on 6p22.1 near the reported locus (6p22.2) for speech measures [55]. Family-based and sib-pair linkage analysis in the candidate gene loci for reading disability confirmed the speech and language-related regions [65]. Rice et al. confirmed the role of multiple genes and shared gene pathways for the comorbidity of language impairment and reading disability [65]. The correlation analysis indicated that 6p22 has a significant correlation between speech measure GFTA and reading measure; Gray Oral Reading Test (GORT) r = 0.657, *p* < 0.01, language measure (PPVT) and GORT r = 0.718, *p* < 0.01, and Omnibus language measure and the reading text comprehension measure (GORT) r = 0.70, *p* < 0.01. The association analysis confirmed the impact of *KIAA0319* on language phenotype [65]. *DCDC2* and *KIAA0319* are present on 6p22, previously reported for dyslexia and childhood apraxia of speech (CAS) [66]. KIAA0319 is a highly expressed protein in the brain and its function is still unknown [67]. At the same time, the DCDC2 is expressed in neuronal precursor cells and plays essential roles in neuronal migration. It contains doublecortin domains that help stabilize and migrate neurons [68]. Our study suggests a linkage at 6p22.1, which provides evidence for the importance of these genes and some other genes present in 6p22 implicated in speech development. 

The two other loci, 14q12 and 16q12.1, were identified in families with SSD and do not overlap with the previously identified loci. The lack of overlap in linked loci might be due to heterogeneity and the use of different assessments in this study. The new suggestive loci should be confirmed in future studies. Despite the high consanguinity rate, our results suggested interfamilial heterogeneity in Pakistani families. It also indicates the polygenic nature of SSD and the involvement of multiple genes in speech acquisition.

## 5. Conclusions

We used locally standardized assessment to assess SSD in our samples, the first essential step to initiate the genetic analysis. The extended consanguineous families in the current study enhanced the utility of genetic research, although with the unknown mode of inheritance and variable disease penetrance in SSD. We confirmed the previously reported SSD locus (6p22.1) and identified new loci (5q21.3, 14q12, and 16q12.1) that may carry genes for SSD and help understand the underlying molecular mechanisms. Utilizing next-generation sequencing (NGS) and finding additional families with overlapping loci in the future is warranted to identify the causative variants in the candidate genes. 

## 6. Limitations

This study is the first to provide essential new genetic loci in Pakistani families and conforming loci reported in other studies. There are a few limitations or future directions that are important to mention. A small number of families in this study cannot confirm the novel loci. Additional families, particularly in this population, will be beneficial in supporting our current findings. We are in the process of enrolling new families, which will further our effort in identifying genes with the mutations causative for SSD and associated language impairment, uncovering the role of shared molecular pathways contributing to these disorders. 

## Figures and Tables

**Table 1:** Speech articulation analysis of PKSLI-94.

Individual IDs	Addition Errors	Omission Errors	Substitution Errors	Total Errors	Correct Consonants	PCC
94001	0	0	10	10	74	88
94002	3	11	44	58	26	31
94003	1	0	2	3	81	96
94004	2	0	7	9	75	89
94006	0	0	14	14	70	83
94007	1	0	19	20	64	76
94008	1	2	33	36	48	57
94009	18	4	56	78	6	7
94010	0	0	0	0	84	100
94011	0	1	3	4	80	95
94012	0	0	4	4	80	95
94013	1	2	19	22	62	74
94014	0	2	2	4	80	95
94015	1	1	5	7	77	92
94016	2	0	22	24	60	71
94017	0	0	2	2	82	98
94018	0	0	1	1	83	99
94019	0	1	5	6	78	93
94020	0	1	6	7	77	92
94021	0	2	13	15	69	82
94022	1	2	3	6	78	93
94025	1	2	23	26	58	69
94026	2	3	25	30	54	64
94027	0	0	0	0	84	100
94028	0	2	9	11	73	87
94029	0	2	16	18	66	79
94030	0	0	1	1	83	99
94031	3	9	18	30	54	64
94032	0	2	8	10	74	88
94033	0	0	28	28	56	67
94034	1	3	15	19	65	77
94035	0	3	2	5	79	94
94036	0	0	4	4	80	95
94037	1	4	4	9	75	89
94038	1	12	41	54	30	36
94039	1	10	37	48	36	43
94046	3	3	32	38	46	55
94047	0	1	2	3	81	96
94058	1	1	7	9	75	89
94062	1	1	10	12	72	86
94063	5	0	25	30	54	64

PCC = Percentage correct consonants. In an Individual ID, the first two digits represent the family number and the next three digits represent the individual who belongs to that family.

**Table 2:** Speech articulation analysis of PKSLI-95.

Individual IDs	Addition Errors	Omission Errors	Substitution Errors	Total Errors	Correct Consonants	PCC
95001	3	3	25	31	53	63
95002	0	1	12	13	71	85
95003	0	1	9	10	74	88
95004	4	4	40	48	36	43
95005	0	1	7	8	76	90
95006	0	0	1	1	83	99
95007	0	0	1	1	83	99
95008	0	1	16	17	67	80
95009	0	0	6	6	78	93
95010	0	0	3	3	81	96
95011	0	1	5	6	78	93
95012	1	1	8	10	74	88
95013	4	5	18	27	57	68
95014	1	0	5	6	78	93
95015	0	0	0	0	84	100

PCC = Percentage correct consonants. In an Individual ID, the first two digits represent the family number and the next three digits represent the individual belonging to that family.

**Table 3:** Speech articulation analysis of PKSLI-97.

Individual IDs	Addition Errors	Omission Errors	Substitution Errors	Total Errors	Correct Consonants	PCC
97001	0	2	21	23	61	73
97002	0	0	1	1	83	99
97003	0	1	7	8	76	90
97004	0	0	1	1	83	99
97005	0	0	14	14	70	83
97006	0	2	3	5	79	94
97007	3	0	6	9	75	89
97008	1	0	6	7	77	92
97009	0	0	12	12	72	86
97010	0	0	0	0	84	100
97011	0	0	0	0	84	100
97012	0	0	8	8	76	90
97013	0	1	9	10	74	88
97014	0	0	1	1	83	99
97015	0	1	14	15	69	82
97016	5	5	20	30	54	64
97017	0	1	11	12	72	86
97018	0	0	4	4	80	95

PCC = Percentage correct consonants. In an Individual ID, the first two digits represent the family number and the next three digits represent the individual belonging to that family.

**Table 4:** Markers with single-point LOD scores in 5q21.3-5q23.1 in PKSLI-97.

Chromosome	SNP ID	hg 19 Position (Mb)	Single-Point LOD Score
5	rs1045706	108.714	−2.157
5	rs6879772	109.613	0.471
5	rs381661	110.085	0.471
5	rs456236	110.087	3.131
5	rs6893213	110.198	0.471
5	rs1837253	110.401	2.689
5	rs10455025	110.404	3.131
5	rs2162893	110.558	0.471
5	rs115192	110.919	2.689
5	rs27342	111.465	2.689
5	rs1850407	112.721	3.131
5	rs26959	112.736	3.131
5	rs1496390	113.725	2.689
5	rs28019	115.733	3.131
5	rs1021516	116.544	0.471
5	rs6891356	116.549	0.471
5	rs13187808	117.223	3.131
5	rs10079352	117.494	3.131
5	rs13157174	117.912	−1.912

**Table 5:** Summary of linkage region and homozygosity mapping in families with SSD.

Linkage Loci (hg 19)	Single-Point LOD Score	Mode of Inheritance	Affection Status	Family/ Branch	Homozygosity
16q12.1-q12.2	2.13	Recessive	Phenotype	PKSLI-94 (Branch 1)	No
6p22.1-p22.1	2.13	Recessive	Phenotype	PKSLI-95	No
14q12-q21.1	2.80	Recessive	Phenotype	PKSLI-95	No
5q21.3-5q23.1	3.13	Recessive	Phenotype	PKSLI-97	Yes

## Data Availability

The data presented in this study are available on reasonable request from the corresponding author.

## Supplementary Materials

The following supporting information can be downloaded at https://www.mdpi.com/article/10.3390/genes15081069/s1, Table S1. Substitution of phonemes at initial, middle, and final positions in words in individuals of PKSLI-94 (Branch1); Table S2. Omission of phonemes at initial, middle, and final positions in words in individuals of PKSLI-94 (Branch 1); Table S3. Addition of phonemes in words in individuals of PKSLI-94 (Branch 1); Table S4. Substitution of phonemes at initial, middle, and final positions in words in individuals of PKSLI-94 (Branch 2); Table S5. Omission of phonemes at initial, middle, and final positions in words in individuals of PKSLI-94 (Branch 2); Table S6. Addition of phonemes in words in individuals of PKSLI-94 (Branch 2); Table S7. Substitution of phonemes at initial, middle, and final positions in words in individuals of PKSLI-95; Table S8. Omission of phonemes at initial, middle, and final positions in words in individuals of PKSLI-95; Table S9. Addition of phonemes at initial, middle, and final positions in words in individuals of PKSLI-95; Table S10. Substitution of phonemes at initial, middle, and final positions in words in individuals of PKSLI-97; Table S11. Omission of phonemes at initial, middle, and final positions in words in individuals of PKSLI-97; Table S12. Addition of phonemes at initial, middle, and final positions in words in individuals of PKSLI-97.
