# Supplementary material for: Whole Genome Analysis in Consanguineous Families Reveals New Loci for Speech Sound Disorder (SSD)
Source: Genes (Basel). 2024 Aug 13;15(8):1069. doi: 10.3390/genes15081069 (PMC11354014; doi:10.3390/genes15081069)
Supplement: Supplementary file 1 [file genes-15-01069-s001.zip › genes-3140582-supplementary.pdf]

Table S1. Substitution of phonemes at initial, middle, and final positions in words in individuals of PKSLI-94 (Branch1)

Consonants	94019	94020	94021	94022	94025	94026	94027	94028	94029	94030	94031	94032	94033	94034	94035	94036	94037	94038	94039
ب[b]	غ (M)					گ (M)		ک (M)	ک (M)				گ (M)						
پ[p]		پھ (F)											ب (I)						
ت[t]						ڈ (I), ک (M), د(F)								و(M)					
ٹ[ʈ]				ڈ (F)	ت (F)	م (I)		ک (I)					ت (I, M), د (F)					ت (M, F)	ت(M, F)
ج[ʤ]					ز (F)					ز(I)		ت (M)	س (F)	ع(F)				ت (F)	د (M), ر(F)
چ[tʃ]						ج(I, M, F)							ت (F)					ت (I, M), تھ (F)	ت(F)
ح[h]													کھ (M)						م(F)
خ[x]	کھ (M)	کھ (M)			ت(I), ٹ(F)	ب(M), ت(F)		کھ(M)	کھ(I, F), د(M)		ت(F)		ک(I), کھ(F)	کھ(M)	کھ(F)	کھ(F)		ت(F)	ک(M), ت(F)
د[d]																			
ڈ[ɖ]	ٹ (F)		ٹ (F)		ٹ(F), د(M)	ٹھ(I)		د(M)				ٹ(F)	د(I, M), ت(F)	ڑ(F)		ٹ(F)		ت(I), د(M)	د(I, M), ن(F)
ر[r]			و(M)		ل(I)	و(M)			ل(I), ڑ(M, F)		ل(I)		ل(I)					ل(I), د(M)	و(M)
ڑ[ɽ]			ر(M)	ڈ(F)	ل(M)						ل(F)		ر(F)	ر(M)			ر(M)		ر(M, F)
ز[z]		ج(M)				ج(M), ب (I)		ج(I)	ج(M)		ج(M)	ج(I)	ج(I, M)				ج(I)	د(M)	د(M)
س[s]						ش(I), چھ(M, F)												ت(I), چھ(M)	ت(I, M, F)
ش[ʃ]						چھ(I)					ت(I), تھ(M), ی(F)	ی(F)	س(M)					ت(I), تھ(F)	ت(I, M, F)
غ[ɣ]	ب(I)	کھ(F)	د(I), ع(M)		د(I, M), ڈ(F)	ر(F)		ف(I)	ب(I)		گ(I), ج(M), کھ(F)	س(F)	ب(I)	د(M, F)			ک(F)	ت(F)	گ (I), گھ(M)
ف[f]				ح(F)		ا(M)		پ(M)	پھ(I)		پھ(I)		ب(I)	پھ(I, M)				پھ(I), پ (F)	پ(I, F), و(M)
ک[k]		کھ(I)	ت (I, M), خ (F)		ت(I), ٹ(M, F)	کھ(F)		ف(F)	گ(F)		کھ(F)		خ(F)	ت(M), کھ(F)	س(F)			ت(I, M, F)	
گ[g]			د(F), ا(I)		د(I, M)	د(F)			د(F)					ر(I)				د(I, M), ٹ(F)	د(F)
ل[l]					ن(M)				ڑ(F)									ر(F)	
م[m]																			
ن[n]																			
و[u]														ب(I)				پ(I), ف(M)	
ی[j/i]		ج(I)											ج(I)	ج(I)		ز(I)	ز(I)	ت(I)	د(I)
بھ[b^h]																		پ(I)	
پھ[p^h]						بھ(I)						ف(I)						پ(I)	پ(I)
تھ[t^h]					ٹھ(F)	ٹھ(F)			ٹھ(F)		ٹھ(F)								ت(I)
ٹھ[ʈ^h]													تھ(I, F)					ت(I), تھ(F)	ت(I), تھ(F)
جھ[ʤ^h]											چ(I)							ت(I)	ت(I)
چھ[tʃ^h]											س(M, F)	ش(M)				ش(M)		چ(I), تھ(M, F)	چ(M), تھ(F)
دھ[d^h]																			
ڈھ[ɖ^h]					د(I)				ٹ(I)				ٹ(I)					ٹ(I)	ت(I)
کھ[k^h]	خ(F)		خ(F), ح(I)		تھ(I, M, F)						چھ(I)		گ(I), خ(M)	چھ(M)				ٹ(I), تھ(F)	
گھ[g^h]			ح(I)		د(I)	ک(I)		ک(I)	ک(I)		ا(I)	ک(I)	ک(I)					ت(I)	ک(I)

I= Initial position, M=Middle position, F= Final position

Table S2. Omission of phonemes at initial, middle, and final positions in words in individuals of PKSLI-94 (Branch 1)

Consonants	94019	94020	94021	94022	94025	94026	94027	94028	94029	94030	94031	94032	94033	94034	94035	94036	94037	94038	94039
ب[b]											ب(M)			ب(F)	ب(F)				ب(M)
پ[p]		پ(I)									پ(I)							پ(F)	
ت[t]			ت(F)					ت(F)			ت(M)								ت(M, F)
ٹ[ʈ]											ٹ(I)						ٹ(F)	ٹ(I)	ٹ(I)
ج[ʤ]																			ج(I)
ح[h]				ح(I)	ح(I)	ح(M)		ح(M)	ح(I, M)		ح(I)	ح(I)						ح(I, M)	ح(M)
خ[x]			خ(F)								خ(I, M)							خ(I, M)	خ(I)
د[d]						د(F)						د(F)					د(F)		د(F)
ڈ[ɖ]											ڈ(F)				ڈ(F)		ڈ(F)	ڈ(F)	
ز[z]											ز(I)							ز(I)	ز(I, F)
ش[ʃ]																		ش(M)	
غ[ɣ]																		غ(I, M)	
ف[f]				ف(M)															
ک[k]	ک(F)																		
گ[g]					گ(F)									گ(M, F)	گ(F)		گ(F)		
کھ[kʰ]						کھ(I)												کھ(I)	

I= Initial position, M=Middle position, F= Final position

Table S3. Addition of phonemes in words in individuals of PKSLI-94 (Branch 1)

Consonants	94019	94020	94021	94022	94025	94026	94027	94028	94029	94030	94031	94032	94033	94034	94035	94036	94037	94038	94039
ا [a]						ا (2)								ا (1)					
ب[b]											ب(1)								ب(1)
ح[h]											ح(1)						ح(1)		
ن[n]											ن(1)								
و[u]				و(1)	و(1)														
ی[j/i]																		ی(1)	

The number in parentheses shows the number of addition of each alphabet.

Table S4. Substitution of phonemes at initial, middle, and final positions in words in individuals of PKSLI-94 (Branch 2)

Consonants	94001	94002	94003	94004	94006	94007	94008	94009	94010	94011	94012	94013	94014	94015	94016	94017	94018	94046	94047	94058	94062	94063
ب[b]	گ(M)	پ(I)												ک(F)				پ(F)		ح(M)		
پ[p]																پھ(I)						
ت[t]		چھ(I), د(F)						ی(M)														د(F), ی(M)
ٹ[ʈ]		ت(M, F)					ت(F)	ت(F), تھ(I)				ت(F)			ت(F)			ت(I, M, F)				ت(I, M, F)
ج[ʤ]								ڈ(M), تھ(F), د(I)														س(F)
چ[tʃ]		ت(F)						تھ(F) ت(I, M)														
ح[h]							ع(M)	ل(M)														
خ[x]	ح(M)	چھ(M)		ت(F)	ت(F), کھ(I, M)	تھ(I, M), ت(F)	ت(I, M, F)	تھ(I,M), ت(F)			ح(M)	تھ(I,M), ت(F)		ح(M)	تھ(I,M), ت(F)	ت(F)		تھ(I), ب(M), ت(F)	ح(M)	کھ(M)	ح(M), ف(F)	ف(M)
د[d]		ٹ(M)									ت(F)											ڈ(F)
ڈ[ɖ]	ٹ(F)	د(I, M), ب(F)	ٹ(F)	ٹ(F)	ٹ(F), د(I)			د(M), ت(F)		ٹ(F)	د(I)			ڑ(F)				د(I, M), تھ(F)		ٹ(F)	ٹ(F)	د(M), ر(I), ل(F)
ر[r]		ل(I), د(M)				ل(F)	ل(I)	ل(I), و(M), د(F)				ل(M)			ل(I, F)			ل(I), د(F)			غ(M)	ل(I), و(M)
ڑ[ɽ]	ل(M)	ر(M, F)		ر(I)	ر(M)	ل(M, F)	ر(M, F)	ل(M, F)				ر(M), ڈ(F)			ل(M, F)			ل(M), ج(F)			ر(M)	ل(M), ر(F)
ز[z]	ج(I, M)	س(I) ج(M)						د(I, M)										د(M), ی(I)		ج(I)		
س[s]		ج(F)						تھ(I, M) چھ(F)														
ش[ʃ]	چھ(I)	س(M, F)					س(I, M, F)	تھ(I, M, F)										س(I, M)				س(I, M)
غ[ɣ]	ب(I)	تھ(I) ر(M)د(F)				د(M, F)	د(I, M) ڈ(F)	د(I, M) ت(F)				د(I, M), ت(F)	ک(F)		د(I, M, F)			ع(I), ل(M), د(F)		ک(I)	ب(I), ر(M), ک(F)	ب(I), کھ(F)
ف[f]		پھ(I) ب(M) و(F)	پھ(I)		پھ(I) پ(F)			پھ(I, F)														
ک[k]	خ(F)	ت(I, M) ٹھ(F)		خ(F)	کھ(F)	ت(I, M, F)	ت(I, M, F)	تھ(I, F), ت(M)				ت(I, M)		گ(I)	ت(I, M, F)			ت(I, M, F)				کھ(F)
گ[g]		د(I), ج(M)		ک(F)		ڈ(I), د(M), ت(F)	د(I, M, F)	د(I, M), ت(F)				د(I, M), ت(F)			د(I, M, F)			د(I, M)			ک(F)	د(F)
ل[l]							ر(M, F)															ر(F)
م[m]		و(M)						پھ(I), ن(M)														
ن[n]		ل(I), و(F)						ل(I)														
و[u]		ل(I)						ف(M)														
ی[j/i]		ل(I)		ز(I)	ز(I)			پ(I)							ج(I)					ج(I)		
بھ[b^{h}]																						
پھ[p^{h}]							پ(I)			ف(I)												
تھ[t^{h}]							ت(I, F)	ت(M)						ت(I)								
ٹھ[ʈ^{h}]		تھ(I, F)			تھ(I)		ٹ(I, F)	تھ(I, F)			ٹ(I)		تھ(I)					تھ(I, F)				تھ(I)
جھ[ʤ^{h}]		ت(I)						ت(I)														
چھ[tʃ^{h}]		تھ(F)			ش(I, M)	ش(M)	س(I, M)	پھ(I), ت(M) , تھ(F)												ش(M)		
دھ[d^{h}]																						
ڈھ[ɖ^{h}]		د(I)						د(I)														د(I)
کھ[k^{h}]		تھ(M, F)				تھ(I, M, F)	تھ(I, M, F)	تھ(I, F) ت(M)				س(I), تھ(M, F)			تھ(I, M, F)			چھ(I, M), تھ(F)			ح(I)	ح(I)
گھ[g^{h}]	ک(I)	ت(I)		ک(I)	ک(I)	د(I)	د(I)	د(I)			خ(I)	د(I)			د(I)		ک(I)	د(I)	ک(I)			

I= Initial position, M=Middle position, F= Final position

Table S5. Omission of phonemes at initial, middle, and final positions in words in individuals of PKSLI-94 (Branch 2)

Consonants	94001	94002	94003	94004	94006	94007	94008	94009	94010	94011	94012	94013	94014	94015	94016	94017	94018	94046	94047	94058	94062	94063
ب[b]		ب(M)																ب(M)				
پ[p]								پ(I)						پ(I)								
ت[t]												ت(F)										
ٹ[ʈ]		ٹ(I)																				
ج[ʤ]		ج(I)																				
ح[h]		ح(M)					ح(I, F)	ح(I, F)				ح(I)						ح(I)	ح(I)	ح(M)		
خ[x]		خ(I, F)																				
د[d]		د(F)						د(F)		د(F)			د(F)									
گ[g]		گ(F)											گ(I)					گ(F)				
ل[l]		ل(M)																				
ن[n]		ن(M)																		ن(F)		

I= Initial position, M=Middle position, F= Final position

Table S6. Addition of phonemes in words in individuals of PKSLI-94 (Branch 2)

Consonants	94001	94002	94003	94004	94006	94007	94008	94009	94010	94011	94012	94013	94014	94015	94016	94017	94018	94046	94047	94058	94062	94063
ا [a]		ا (2)	ا (1)	ا (1)		ا (1)	ا (1)	ا (2)				ا (1)										ا (3)
پ[p]																		پ(1)				
ح[h]								ح(1)						ح(1)	ح(2)							
ن[n]																		ن(2)			ن(1)	ن(2)
و[u]				و(1)				و(2)												و(1)		
ی[j/i]		ی(1)						ی(12)														
پھ[p^h]								پھ(1)														

The number in parentheses shows the number of addition of each alphabet.

Table S7. Substitution of phonemes at initial, middle, and final positions in words in individuals of PKSLI-95

Consonants	95001	95002	95003	95004	95005	95006	95007	95008	95009	95010	95011	95012	95013	95014	95015
ب[b]	گ (M)				گ (M)								گ (M)		
پ[p]		م (F)	ش (I)					پھ (I)						پھ (F)	
ت[t]															
ٹ[ʈ]	ت (I, M, F)		ت (F)	ت (I, M, F)				ٹھ (F)			ٹھ (F)	ت (I)	ت (F)		
ج[ʤ]	چ (F)			ل (I), د (M), ت(F)				چ (I)					ت (F)		
چ[tʃ]	ن (M)			ش (F), ت (I, M)	ت (M)										
ح[h]				ب (F)											
خ[x]	کھ(M), ح (F)	کھ (M, F)	کھ (M, F)	گ (M), ح (F)	کھ (M), ت (F)		کھ (F)	پھ (M), کھ (F)	کھ (M, F)	کھ (M, F)	ح (F)	کھ (M), ک (F)	ب (M), ٹ (F)	ح (F, M)	
د[d]				ت (F)	ت (F)							ڈ (F)	ت (F)		
ڈ[ɖ]	د (I, M), ٹ (F)	ت (F)	ٹھ (F)	د (I, M), ٹ (F)		ٹ (F)		ٹھ (F)		ڑ (F)			ٹھ (F)		
ر[r]	و(M)	ڑ (M)	ڑ (I)	ل (I), غ (M), ش (F)				ڑ (M), ش (F)			ڑ (M)		ل (I), ڑ (M), ی (F)		
ڑ[ɽ]	ر(M)	ر (M, F)		تھ (M), ر (F)	ر (M)			ر (M, F)	ر (M, F)		ر (M)	ر (M)	ر (M)		
ز[z]	و(M)			گ (I), ر (M)											
س[s]				چھ (F)											
ش[ʃ]	چھ(I), س(F)			تھ (M), س (F)									ج (M)		
غ[ɣ]	ب (I), کھ(F)			ر (M)									ل (F)		
ف[f]				چ (I)										پھ (I)	
ک[k]	کھ (F)	کھ (F)	کھ (F)	کھ (F)				کھ (F)	کھ (F)		کھ (F)			کھ (F)	
گ[g]	د (F)	کھ (F)	ک (I)												
ل[l]				ب (M)				ٹ (I)							
م[m]	ن (M)														
ن[n]				تھ (M), م(F)											
و[u]															
ی[j/i]								ش (I)							
بھ[b^{h}]								پھ (I)							
پھ[p^{h}]				پ (I)											
تھ[t^{h}]	د (I), ت (F)	ٹ (I)	د (I)	ت (M)					ت (F)			ٹ (I, M, F)	ٹ (I), ت (F)		
ٹھ[ʈ^{h}]		تھ (I)		ت (I), تھ (F)	تھ (I)			تھ (I)					ت (I)		
جھ[ʤ^{h}]				د (I)											
چھ[tʃ^{h}]	س (F)			تھ (M), ت (F)											
دھ[d^{h}]															
ڈھ[ɖ^{h}]	دھ (I)												دھ (I)		
کھ[k^{h}]		ک (M)		جھ (I)									ج (M)		
گھ[g^{h}]								کھ (I)							

I= Initial position, M=Middle position, F= Final position

Table S8. Omission of phonemes at initial, middle, and final positions in words in individuals of PKSLI-95

Consonants	95001	95002	95003	95004	95005	95006	95007	95008	95009	95010	95011	95012	95013	95014	95015
پ[p]				پ (I)											
ت[t]	ت (M, F)											ت (F)			
ٹ[ʈ]													ٹ (I)		
چ[tʃ]		چ (M)													
ح[h]													ح (M)		
خ[x]				خ (I)									خ (I)		
د[d]	د (F)			د (M)							د (F)				
ڈ[ɖ]					ڈ (F)										
ر[r]				ر (F)											
ز[z]			ز (M)												
گ[g]								گ (F)							
ی[j/i]													ی (I)		
کھ[k^h]													کھ (F)		

I= Initial position, M=Middle position, F= Final position

Table S9. Addition of phonemes at initial, middle, and final positions in words in individuals of PKSLI-95

Consonants	95001	95002	95003	95004	95005	95006	95007	95008	95009	95010	95011	95012	95013	95014	95015
ا [a]	ا (1)			ا (1)									ا (1)		
ب[b]	ب (1)														
ح[h]													ح (1)		
ر[r]				ر (1)									ر (1)		
ل[l]				ل (1)											
ن[n]	ن (1)											ن (1)		ن (1)	
ی[j/i]				ی (1)											

The number in parentheses shows the number of addition of each alphabet.

Table S10. Substitution of phonemes at initial, middle, and final positions in words in individuals of PKSLI-97

Consonants	97001	97002	97003	97004	97005	97006	97007	97008	97009	97010	97011	97012	97013	97014	97015	97016	97017	97018
ب[b]								گ(F)							و (F)	و (F)		
پ[p]			ک (F)												ف (F)			
ت[t]																ک (M)		
ٹ[ʈ]	ت (I, M, F)				ت (I, M, F)				ت (I, M, F)						ت (I, M, F)	ت (M), ٹھ (F)	ت (I, M, F)	
ج[ʤ]	چ (I)												ح (F)		ت (F)	س(F)		
چ[tʃ]														چھ (F)				
ح[h]																کھ (F)		
خ[x]	ت (F), کھ (M)		ت (F)		ت (F)		کھ (M, F)	ت (F)	کھ (M, F)				ک (M)		کھ (M)	کھ (F)		ت (F)
د[d]	ت (F)																	
ڈ[ɖ]	د (I), ن (F)	ٹ (F)	ٹ (F)		د (I, M), ت (F)			ٹ (F)	د (I, M)			ٹ (F)	ٹ (F)		د (I, M)	ٹھ (F)	د (I, M), ت (F)	
ر[r]			ڑ (M)	ڑ (M)	ڑ (M)											و (M)		
ڑ[ɽ]	ر (M, F)		ر (M, F)		ر (M, F)	ر (M, F)	ر (M)	ر (M)	ر (M)			ر (F)	ر (M)		ر (M, F)	ر (F)	ر (M)	
ز[z]	ی (I, M)											ی (I, M)	ج (I,M)			ب (I)		ج (I)
س[s]	ش (I, M, F)															چ (I)		
ش[ʃ]												چھ (I)	چھ (I)			چ (I), ج (M)		
غ[ɣ]							کھ (M)											
ف[f]												پھ (I)						پھ (I)
ک[k]									کھ (F)			خ (F)						
گ[g]	د (F)		ک (F)				کھ (F)	کھ (F)								ک (F)		
ی[j/i]																د (I)		
تھ[t^h]					ت (M)		ت (I)	د (I)								ت (I), کھ (F)		
ٹھ[$ʈ^h$]	تھ (I, F)				تھ (I, F)	ٹ (I)			تھ (I, F)				تھ (I)		تھ (I, F)		تھ (F), کھ (I)	
چھ[$tʃ^h$]	ش (F)																	
ڈھ[$ɖ^h$]					دھ (I)				دھ (I)						دھ (I)	دھ (I)	دھ (I)	
کھ[k^h]	ج (I)												خ (F)			غ (I)		خ (F)
گھ[g^h]												ک (I)					ک (I)	

I= Initial position, M=Middle position, F= Final position

Table S11. Omission of phonemes at initial, middle, and final positions in words in individuals of PKSLI-97

Consonants	97001	97002	97003	97004	97005	97006	97007	97008	97009	97010	97011	97012	97013	97014	97015	97016	97017	97018
ب[b]																ب (M)	ب (M)	
ت[t]						ت (F)							ت (F)					
ٹ[ʈ]																ٹ (F)		
ح[h]			ح (I)													ح (M)		
خ[x]	خ (I)															خ (I, M)		
د[d]						د (F)												
ڈ[ɖ]															ڈ (F)			
غ[γ]	غ (F)																	

I= Initial position, M=Middle position, F= Final position

Table S12. Addition of phonemes at initial, middle, and final positions in words in individuals of PKSLI-97

Consonants	97001	97002	97003	97004	97005	97006	97007	97008	97009	97010	97011	97012	97013	97014	97015	97016	97017	97018
ح[h]							ح (1)											
ک[k]																ک (1)		
ن[n]							ن (2)	ن (1)								ن (4)		

The number in parentheses shows the number of addition of each alphabet
